# Acute Stress, Induced by IFNγ  + Aβ, and Chronic Stress, Induced by Age, Affect Microglia in a Sex-Specific Manner

**DOI:** 10.1007/s12035-023-03235-9

**Published:** 2023-02-14

**Authors:** Virginia Mela, Aline Sayd Gaban, Paul Marie Shatz, Marie-Victoire Guillot-Sestier, Marina A. Lynch

**Affiliations:** 1 Trinity College Institute of Neuroscience, Trinity College Dublin, Dublin, Dublin 2 Ireland; 2 Present Address: Department of Medicine and Dermatology, Faculty of Medicine, University of Malaga, Malaga, 29010 Spain; 3 Present Address: School of Medicine and Conway Institute, University College Dublin, Dublin, Ireland

**Keywords:** Microglia, Metabolism, Age, Phagocytosis, Microglial motility

## Abstract

Microglial phenotype changes in the aged brain, and also in neurodegenerative diseases, and it is generally accepted that these changes at least contribute to the inflammation that can have detrimental effects on brain health. Accumulating data have determined that there are multiple microglial activation states with consistent findings indicating that with stressors including age, a switch towards an inflammatory phenotype occurs. Among the changes that accompany this is a change in metabolism, whereby glycolysis is increased in microglia. Here, we asked whether sex impacted on the response of microglia to two stressors, interferon-γ + amyloid-β (IFNγ  + Aβ) and age. The data show that IFNγ  + Aβ triggered cells from female mice to adopt a glycolytic phenotype. Metabolism was also altered with age; microglia from aged male mice responded by increasing oxidative phosphorylation, and microglial motility was preserved, contrasting with microglia from female mice where motility was compromised. We conclude that sex is a significant variable in the responses of microglia to stressors.

**Supplementary Information:**

The online version contains supplementary material available at 10.1007/s12035-023-03235-9.

## Introduction

The multiple functions and phenotypes of microglia are well rehearsed as a consequence of the significant research focus on microglial biology in the past two decades. However, despite the marked progress, there are numerous outstanding issues to be addressed, including a more precise understanding of how microglia contribute to age-related and neurodegenerative changes. A great body of research has coalesced to establish that the primary role of microglia is neuroprotective and this is supported by functions such as motility and phagocytosis, whereby microglia react quickly to respond to potential threats [1]. With the caveat that there is an ongoing need for precision in describing microglia, it seems likely that uncontrolled microglial activation is damaging, and likely to contribute to disease-related and age-related compromise in neuronal function [1, 2]. Certainly RNAseq analysis has revealed marked changes in microglia isolated from young and aged mice with downregulation of transcripts that describe homeostatic microglia and upregulation of transcripts that code for proteins associated with the inflammatory response [3–6]. Furthermore, reports from several groups identify several consistent age-related changes in microglia including responsiveness to inflammatory stimuli [7–9] and the consequent secretate [10, 11]. Morphological changes [12] as well as functional changes [13] have also been reported.

The concept of microglial activation is becoming ever more complex with evidence demonstrating the importance of context [14, 15] and with omics data suggesting the existence of multiple activation states [16]. A further layer of complexity has been added with the evidence that microglial metabolism, which shifts as cell activation state changes, is a significant player in maintenance of cell function [13, 17] and that sex-related differences in microglia, which are well documented in development and early life [18, 19], persist into adulthood and beyond [20].

Here, we set out to evaluate whether sex impacts on the response of microglia to 2 different stressors, exposure to interferon-γ  + amyloid-β (IFN γ  + Aβ) in vitro, and age. The data indicate that sex is an important determinant of the metabolic response of cells to these stressors and this impacts on cell function. In the context of age, the evidence suggests that microglia from male mice are less vulnerable than cells from female mice and are capable of responding to age by increasing oxidative metabolism and maintaining cell motility. The data highlight the importance of including sex as a variable in analysis of age-related changes in the brain.

## Materials and Methods

### Animals

Female and male young and aged (4–5-month and 17–22-month-old) C57BL/6 mice, and neonatal C57BL/6 mice, were used in these experiments. All experiments were performed under license from the Health Products Regulatory Authority of Ireland in accordance with EU regulations and with local ethical approval (Trinity College Dublin). Animals were housed under controlled conditions (20–22 °C, food and water ad lib) and maintained under veterinary supervision.

### Preparation of Primary Microglia from Neonatal Mice

Microglia were prepared as previously described [21]. Briefly, mixed glia were isolated from triturated and filtered cortical tissue of C57BL/6 neonatal mice. Tissue was centrifuged (800 × *g*, 5 min), the pellet was resuspended in pre-warmed media, triturated and cells were seeded (1 ml) and cultured in T25 cm^2^ flasks in cDMEM (31,330–038, Bioscience, USA) supplemented with macrophage colony stimulating factor (M-CSF; 100 ng/ml; 416-ML, R&D Systems, UK), and granulocyte macrophage colony stimulating factor (GM-CSF; 100 ng/ml; 415-ML, R&D Systems, UK) for 10–12 days with media change every 3–4 days. Cells were shaken for 2–3 h (37 °C) to dislodge non-adherent microglia and, after centrifuging (800 × *g*, 5 min), the pellet was harvested, cells counted, seeded in 24-well plates (1 × 10^5^ cells/well), and cultured for a further 2 days.

Cells were incubated for 24 h in the presence or absence of IFNγ (12.5 ng/ml) + and amyloid-β (Aβ; 10 μM comprising 4.8 μM Aβ_1–40_ and 5.8 μM Aβ_1–42_; Invitrogen, USA). Aβ was prepared as follows: lyophilised Aβ_1–40_ and Aβ_1–42_ peptides were dissolved in HPLC grade water to give a stock solution (6 mg/ml), which was diluted using sterile PBS (final concentration 1 mg/ml). This was allowed to aggregate (24 h, 220 rpm, 37 °C) to give a mix that was shown, by enhanced Thioflavin T binding, to contain both oligomers and fibrils (Supplementary Fig. 1).

### Isolation of Microglia from Young and Aged Mice

Tissue from whole brain of young and aged mice was homogenised using the gentle-MACS Dissociator (Miltenyi Biotec, UK) in combination with the AdultBrain Dissociation Kit (130–107-677; Miltenyi Biotec UK); filtered through a MACS SmartStrainer (70 µm); washed with D-PBS containing PBS, calcium (100 mg/l), magnesium (100 mg/l), glucose (1000 mg/l) and pyruvate (36 mg/l), and centrifuged (3000 × g; 10 min, 4 °C) yielding a pellet that contained the microglia as described [22]. Pellets were resuspended in cold D-PBS (3.1 ml), debris removal solution (900 µl) was added, and the suspension was gently overlaid with cold D-PBS (4 ml) and (3000 × g, 4 °C, 10 min). The third phase that contained microglia was resuspended in cold D-PBS (15 ml) and centrifuged (1000 × g, 10 min, 4 °C), and pellets were resuspended in red blood cell removal solution (1 ml) and incubated (10 min, 4 °C). After addition of cold D-PBS (10 ml), samples were centrifuged (300 × g, 10 min, 4 °C) and the pellet was resuspended in D-PBS; microglia were incubated with CD11b microbeads (130–126-725; Miltenyi Biotec, UK), magnetically separated using the QuadroMACS separator (Miltenyi Biotec, UK), and resuspended in PB buffer (PBS + 0.5% foetal bovine serum (10,270–106, Bioscience, USA)). Samples were centrifuged (300 × g; 10 min), the final microglial pellet was resuspended in PBS (75 μl), and cells were seeded in 48-well plates (2.5 × 10^4^ cells/well; final volume 200 μl) and cultured in cDMEM supplemented with M-CSF (100 ng/ml; 416-ML, R&D Systems, UK) and GM-CSF (100 ng/mL; 415-ML, R&D Systems, UK) for 5 days with a change of media on alternate days. At the time of seeding, purity of the microglia isolated was assessed by flow cytometry in aliquots stained with a Hoechst/Propidium Iodide Stain (ThermoFisher Scientific, 62249, P3566), PE/Cy7-CD11b (Biolegend, 101215), and Alexa-fluor-647-GFAP (BD Bio, 561470) antibodies. At the time of seeding, the purity of the microglia isolated with magnetic-CD11b beads was routinely assessed by flow cytometry. Typically, > 95% of live cells were CD45^+^, CD11b^+^, and GFAP^−^ (Supplementary Fig. 2).

### Analysis of ECAR and OCR

The SeaHorse Extracellular Flux (XF96) Analyser (SeaHorse Bioscience, USA) was used to assess the ECAR in cultured microglia from neonatal mice and OCR in microglia isolated from young and aged mice. For analysis of ECAR, microglia (6 × 10^4^ cells/well) were seeded (180 μl/well) on SeaHorse cell culture microplates (101,085–004, SeaHorse Bioscience, USA) and treated with IFNγ (12.5 ng/ml) + Aβ (10 μM) as above. SeaHorse XF Calibrant solution (200 μl; 100,840–000, SeaHorse Bioscience, USA) was added to each well of the utility plate which was kept overnight in a CO_2_-free incubator at 37 °C. Cells were washed with assay medium (200 μl) in accordance with the manufacturer’s instructions, and samples were incubated in assay media (final volume, 200 μl/well; 37 °C; 1 h) in a CO_2_-free incubator. Glucose (10 mM), oligomycin (20 μM), and 2-deoxy-D-glucose (2-DG; 500 mM; all Sigma-Aldrich, UK) were prepared in glycolytic flux assay media and loaded into the appropriate ports for sequential delivery to the sample wells. Following calibration, ECAR was measured every 8 min for 96 min during which time the appropriate compounds were injected at 24-min intervals. ECAR was automatically calculated using the SeaHorse XF96 software; 3–5 replicates for adults and 3 for neonates were assessed for each separate sample.

OCR was assessed in microglia from young and aged male and female mice using the mitochondrial stress test. Microglia (6 × 10^4^ cells/well) were seeded (180 μl/well) on SeaHorse cell culture plates, and the sensor cartridge was hydrated as described above by adding SeaHorse XF Calibrant solution (200 µl; 100,840–000, SeaHorse Bioscience, USA) to each well of the utility plate and left overnight in a CO_2_-free incubator at 37 °C. Cells were washed twice with assay medium (90 µl), and cells were incubated (37 °C, 1 h) in a CO_2_-free incubator in a final volume of 180 μl/well. Oligomycin (20 μM; AbCam, UK), carbonyl cyanide-4-(trifluoromethoxy)phenylhydrazone (20 μM; FCCP; Sigma-Aldrich, UK), and antimycin A/rotenone (40 μM; Sigma-Aldrich, UK) were loaded into the appropriate ports and, after calibration, delivered sequentially. These events allow OCR to be measured every 8 min for 96 min (at 24min intervals). Oligomycin is a complex V inhibitor and allows ATP-linked respiration to be assessed (by subtracting basal OCR from the value following oligomycin). FCCP collapses the inner membrane gradient maximising electron function and allows assessment of maximal respiratory capacity (by subtracting non-mitochondrial respiration from the FCCP rate) and antimycin A/rotenone (40 μM; Sigma-Aldrich, UK) which inhibit complexes III and I, shuts down the electron transport chain, and therefore collapses mitochondrial respiration. OCR was automatically calculated using the SeaHorse XF96 software, and 3–5 replicates were assessed for each separate sample.

### Assessment of PFKFB3 by Gel Electrophoresis

Microglia were prepared for western immunoblotting as previously described [23]. Briefly, cells were incubated in lysis buffer (composition in mM: Tris–HCl 10, NaCl 50, Na_4_P_2_O_7_.H_2_O 10, NaF 50, containing 1% each of Igepal, phosphatase inhibitor cocktails I and II, and protease inhibitor; Sigma, UK), equalised for protein, added to 4 × SDS sample buffer (composition: Tris–HCl 100 mM, pH 6.8, 4% SDS, 2% bromophenol blue, 20% glycerol; Sigma, UK), and boiled (95 °C, 5 min).

Samples were applied to 10–15% SDS gels, proteins were transferred to PVDF membrane, non-specific binding was blocked (5% Marvel in TBS containing 0.05% Tween 20), and membranes were incubated overnight at 4 °C with rabbit anti-PFKFB3 antibody (1:750 in 5% non-fat dried milk/TBS-T; ab181861, AbCam, USA). Membranes were washed and incubated (room temperature, 2 h) with a secondary HRP-linked anti-rabbit antibody (1:2000 in 5% milk in TBS-T; 111–035-003, Jackson, USA)). Bands were detected using WesternBright ECL chemiluminescent substrate (Advansta, USA) and images (Fujifilm LAS-4000 imager) evaluated using ImageJ (http://rsb.info.nih.gov/).

### Uptake of Fluorescently Labelled Beads

Uptake of latex beads (1.0 μm mean particle size; L4655, Sigma-Aldrich, Ireland) in a fluorescent yellow-green aqueous suspension was used to analyse phagocytic capacity in microglia isolated from young and aged mice. Cells, prepared as described above, were incubated (4 h) with 0.025% latex beads in fresh cDMEM media. Cells were washed, fixed in 4% PFA, and washed in PBT (PBS + 1% Triton X-100) prior to staining. Cells were blocked (1 h; PBT + 3% BSA), incubated with rabbit anti-Iba1 (19–7141, Wako, Japan 1:1000; overnight; 4 °C), washed, incubated with Alexa Fluor® 546 donkey anti-rabbit IgG (1:1000; 2 h; room temperature, A10040, Invitrogen, USA), and mounted in ProLong Gold with DAPI (P36941, Thermo Scientific, USA). Images (8 fields; 40 × magnification) were acquired with a Zeiss AX10 Imager A1 microscope. Analysis of images was undertaken with the ImageJ software, and the number of latex beads^+^ microglia was assessed.

### Analysis of Phagocytosis of Synaptosomes

Brain tissue was homogenised (10% weight/volume 0.32 M sucrose in HEPES; pH 7.4) and centrifuged (5 min, 5000 rpm, 4 °C) to yield supernatant which was collected and centrifuged (15 min, 14,000 rpm, 4 °C) to provide a synaptosome-enriched pellet which was resuspended in complete culture media (DMEM-F12, FBS 0.5%, penicillin/streptomycin 50 U/ml (15,070–063, Biocience, USA)). Microglia (60,000 cells per well on coverslips in 48-well plates) were incubated (24 h) with synaptosomal preparations (final concentration 1 µg/µl), washed × 3 in complete culture media (1 ml), rinsed in PBS, fixed in PFA (4%, 20 min, RT), and washed before staining.

Coverslips were incubated (1 h, RT) in PBS containing Triton (0.1%) and horse serum (10%), incubated (2 h, RT) with anti-Iba1 (1:1000; 19–7141, Wako) and anti-CD68 (1:100; MCA1957, AbD Serotec) and thereafter with secondary antibodies (1 h, RT; donkey anti-rabbit 1:1000, A10040, Invitrogen for Iba1, and goat anti-rat 1:1000, A21094, Invitrogen for CD68). Coverslips were then incubated with anti-synaptophysin (1:100; overnight, 4 °C; S5748, Sigma UK) and secondary antibody (donkey anti-mouse 1:1000, A21202, Invitrogen; 1 h, RT) with washes between incubations. Coverslips were mounted on slides using Vectashield antifade mounting medium with Dapi (H-1500–10, Vector laboratories) and observed under a confocal microscope (Leica SP8). To quantify synaptosomal engulfment by microglia, z-stacks were taken with a 63 × oil objective. Quantitative 3D in silico modelling (q3DISM) of synaptosome uptake by microglia was adapted from a method previously developed using the Imaris BitPlane software (version 9.1) [24].

### Analysis of Microglial Motility

To assess motility of microglia prepared from young and aged mice, we used the wound healing assay. Microglia were cultured on inserts in 48-well plates for 5 days. Two scratches at right angles were drawn using a 10 ml pipette tip to create “the wound.” Images were taken using an Olympus IX51 light microscope with a built-in camera (Olympus, Japan) 8 h after the scratch and the width of the scratches were measured to quantify the distance and speed travelled by the cells.

### PFKFB3 Staining

Microglia, prepared as described above, were washed, permeabilised (10 min; PBS with 0.1% Triton-X100), blocked (1 h; 3% BSA with 0.1% Triton-X100), and incubated in the presence of rabbit anti-PFKFB3 (1:250; ab181861, Abcam, UK) and goat anti-Iba1 (1:750; LS-B2645-50, LSBio Inc., USA; overnight; 4 °C) and thereafter with Alexa Fluor® 546 donkey anti-goat IgG (1:1000, A11056, Invitrogen) and Alexa Fluor® 488 donkey anti-rabbit IgG (1:1000; A21206, Invitrogen, 2 h, room temperature). Coverslips were mounted in ProLong®Gold with the nuclear marker DAPI (P36941, ThermoScientific, USA); images were captured using a Leica SP8 scanning confocal microscope (40 × ; 5 fields of view) and processed using the ImageJ software. Total cellular fluorescence (minus DAPI) was corrected by subtracting the product of the area of selected cell fluorescence and the mean background fluorescence from the integrated density as previously described [17].

### PFKFB3 mRNA

RNA, isolated from cells using a Nucleospin® RNAII kit (Macherey–Nagel GmbH, Germany), was reverse transcribed into cDNA using a high-capacity cDNA Archive kit (Applied Biosystems, UK), and real-time PCR was performed on duplicate samples with predesigned Taqman gene expression assay for PFKFB3 (Mm00504650_m1) using an ABI Prism 7300 instrument (Applied Biosystems, UK); the endogenous control was β-actin, which was used to normalise gene expression data. Gene expression was calculated relative to the endogenous control samples and to the control sample giving an RQ value (2^−DDCt^, where CT is the threshold cycle).

### Statistical Analysis

Data are reported as the mean ± SEM, and the number of experiments is indicated in each case. Statistical analysis was carried out using Student’s *t*-test for independent means, or 2-way ANOVA followed by post hoc Tukey’s multiple comparisons test, as appropriate. The significance level was set at *p* < 0.05.

## Results

**Fig. 1:**
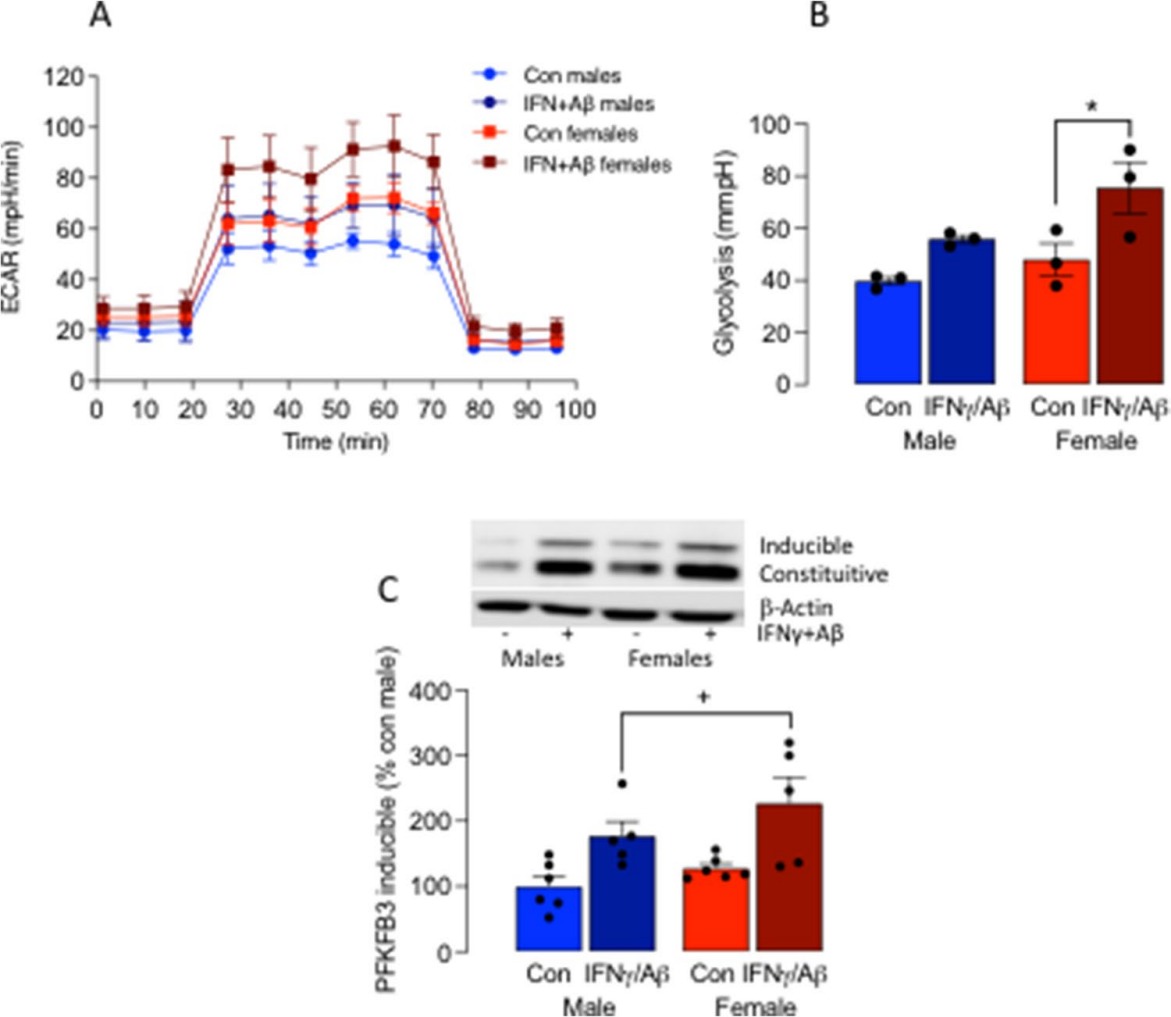
The effect of IFNγ  + Aβ on microglia is sex-dependent. Microglia, prepared from male and female neonatal mice (1-day-old), were cultured and treated with IFNγ  + Aβ as described in “Materials and Methods.” Cells were assessed for metabolic profile using Seahorse technology and for expression of PFKFB3 by gel electrophoresis and immunoblotting. **A** IFNγ  + Aβ increased ECAR to a greater extent in microglia from female mice. **B** Analysis of the mean data identified a significant main effects of treatment (*p* < 0.01) and sex (*p* < 0.05) on glycolysis and post hoc analysis indicated that IFNγ  + Aβ significantly increased glycolysis in microglia from female mice only (*p < 0.05). **C** Analysis of the effect of IFNγ  + Aβ on PFKFB3 revealed a significant main effect of sex (*p* < 0.01), while post hoc analysis indicated a significant increase in the IFNγ  + Aβ-induced change in microglia from female mice compared with males (*p < 0.05). Data are means ± SEM (3 replicates for 3 neonates/group in separate experiments)

IFNγ  + Aβ increased ECAR in microglia prepared from neonatal male and female mice compared with controls (Fig. 1A) although the effect was more marked in cells from female mice. Analysis of the mean data indicated that there was a main effect of treatment (*p* < 0.01) and sex (*p* < 0.05); post hoc analysis indicated that glycolysis was significantly increased in microglia from IFNγ  + Aβ-treated female mice compared with control-treated female mice (*p < 0.05; Fig. 1B) but that the IFNγ  + Aβ-related increase in cells from male mice failed to reach statistical significance. PFKFB3 is a key glycolytic enzyme that is closely correlated with glycolysis in microglia [13, 17, 22], and here, analysis of the effect of IFNγ + Aβ revealed a significant main effect of sex (*p* < 0.01; Fig. 1C), while post hoc analysis indicated that it was significantly increased in IFNγ  + Aβ-treated microglia from female mice compared with IFNγ  + Aβ-treated microglia from male mice (*p < 0.05; Fig. 1C).

**Fig. 2:**
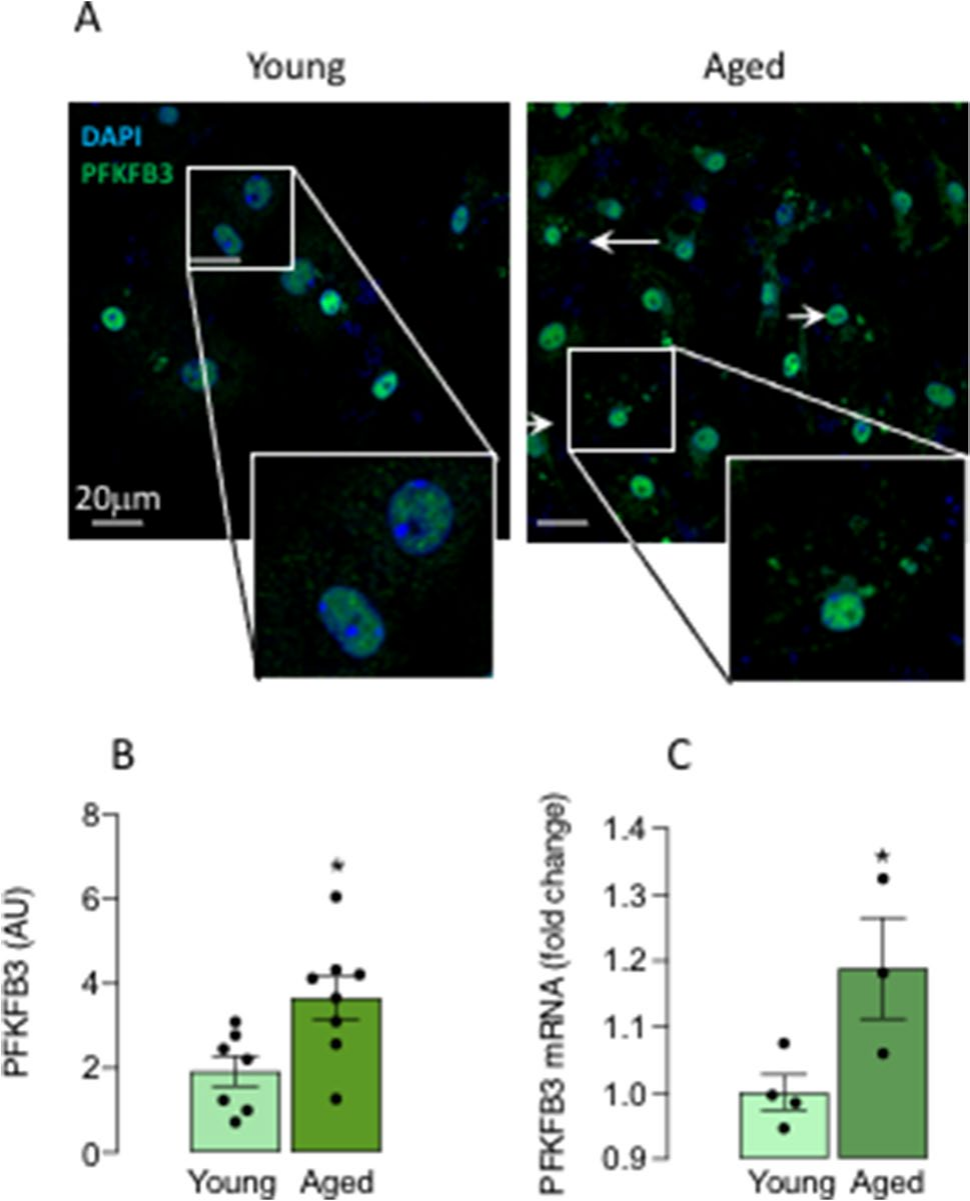
PFKFB3 is increased with age. Microglia were prepared from young (4–5-month-old) and aged (17–22-month-old) mice as described in “Materials and Methods”. **A** PFKFB3 staining in microglia isolated from the brain of young mice co-localised mainly with DAPI-stained nuclei. Cytosolic PFKFB3 staining (**B**) and PFKFB3 mRNA expression (**C**) were significantly increased with age (*p < 0.05). Data are expressed as the mean ± SEM (*n* = 7 or 8 for PFKFB3 staining and *n* = 4 or 5 for PFKFB3 mRNA)

**Fig. 3:**
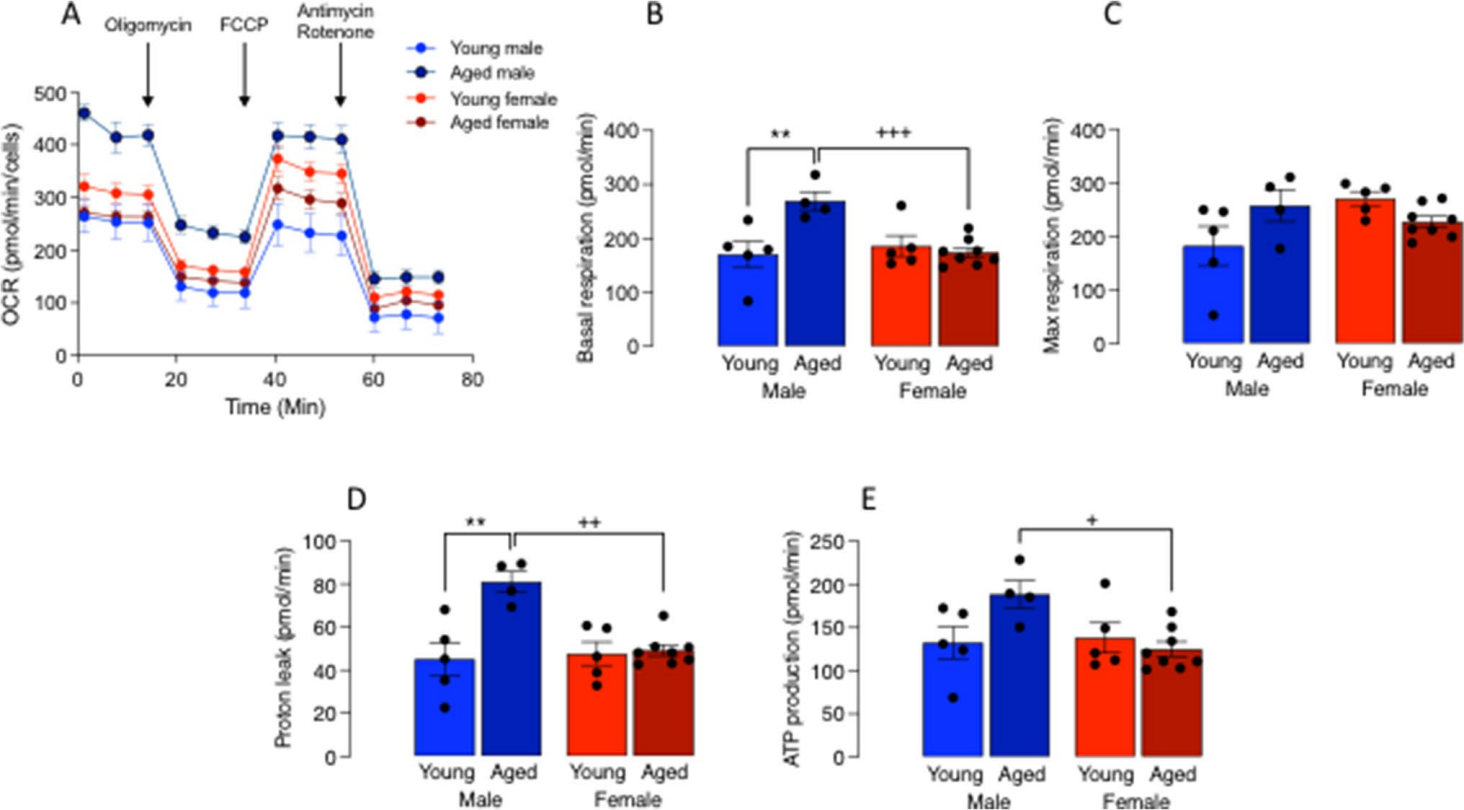
Age-related metabolic change in microglia are sex-specific. Microglia were prepared from young (4–5-month-old) and aged (17–22-month-old) mice as described in “Materials and Methods.” **A** The metabolic profile (expressed as OCR (pmol/min/cells)) is presented following sequential addition of oligomycin, FCCP, and antimycin A/rotenone as indicated by the arrows and as described in “Materials and Methods.” OCR was increased in microglia from male mice. Data are derived from 3 to 5 replicates for 3 adults/treatment group and expressed as the mean ± SEM. Significant sex × age interactions were observed in basal respiration (**B**; *p* < 0.01), maximal respiration (**C**; *p* < 0.05), proton leak (**D**; *p* < 0.01), and ATP production (**E**; *p* < 0.05). Basal respiration and proton leak were significantly increased in microglia from aged, compared with young males (**p < 0.01), and basal respiration, proton leak, and ATP production were significantly reduced in cells from aged female mice compared with aged male mice (^+^*p* < 0.05; ^++^*p* < 0.01; ^+++^*p* < 0.001). Data are expressed as the mean ± SEM (*n* = 4–8)

We then wanted to determine whether microglia responded in a sex-related manner to another stressor, age, and therefore, we assessed microglial metabolism and function in isolated cells prepared from young and aged mice. First, without regard to sex, we assessed PFKFB3 staining in microglia prepared from young (4–5-month-old) and aged (17–22-month old) mice and show that it was largely co-localised with DAPI-stained nuclei in cells from young mice, whereas it translocated from the nucleus to the cytosol in cells from aged mice (Fig. 2A) so that cytosolic, active PFKFB3 was significantly increased with age (*p < 0.05; Fig. 2B). Similarly, PFKFB3 mRNA expression was increased in microglia from aged, compared with young, mice (*p < 0.05; Fig. 2C) confirming our previous results [13] and consistent with the idea that age stimulates microglia to switch their metabolism towards glycolysis [13]. This opens the possibility that mitochondrial metabolism may be affected with age; therefore, we proceeded to assess OCR in microglia from young and aged mice and, in this case, evaluated the impact of sex. The data identified sex-related differences in metabolic profile in microglia (Fig. 3A). A significant sex × age interaction was observed in basal respiration (*p* < 0.01; Fig. 3B), maximal respiration (*p* < 0.05; Fig. 3C), proton leak (*p* < 0.01; Fig. 3D), and ATP production (*p* < 0.05; Fig. 3E). Post hoc analysis indicated that there was a significant increase in basal respiration (**p < 0.01) and proton leak (**p < 0.01) in microglia from aged compared with young male mice, and a significant decrease in basal respiration (^+++^*p* < 0.001), proton leak (^++^*p* < 0.01), and ATP production (^+^*p* < 0.05) in cells from aged female mice compared with aged male mice.

**Fig. 4:**
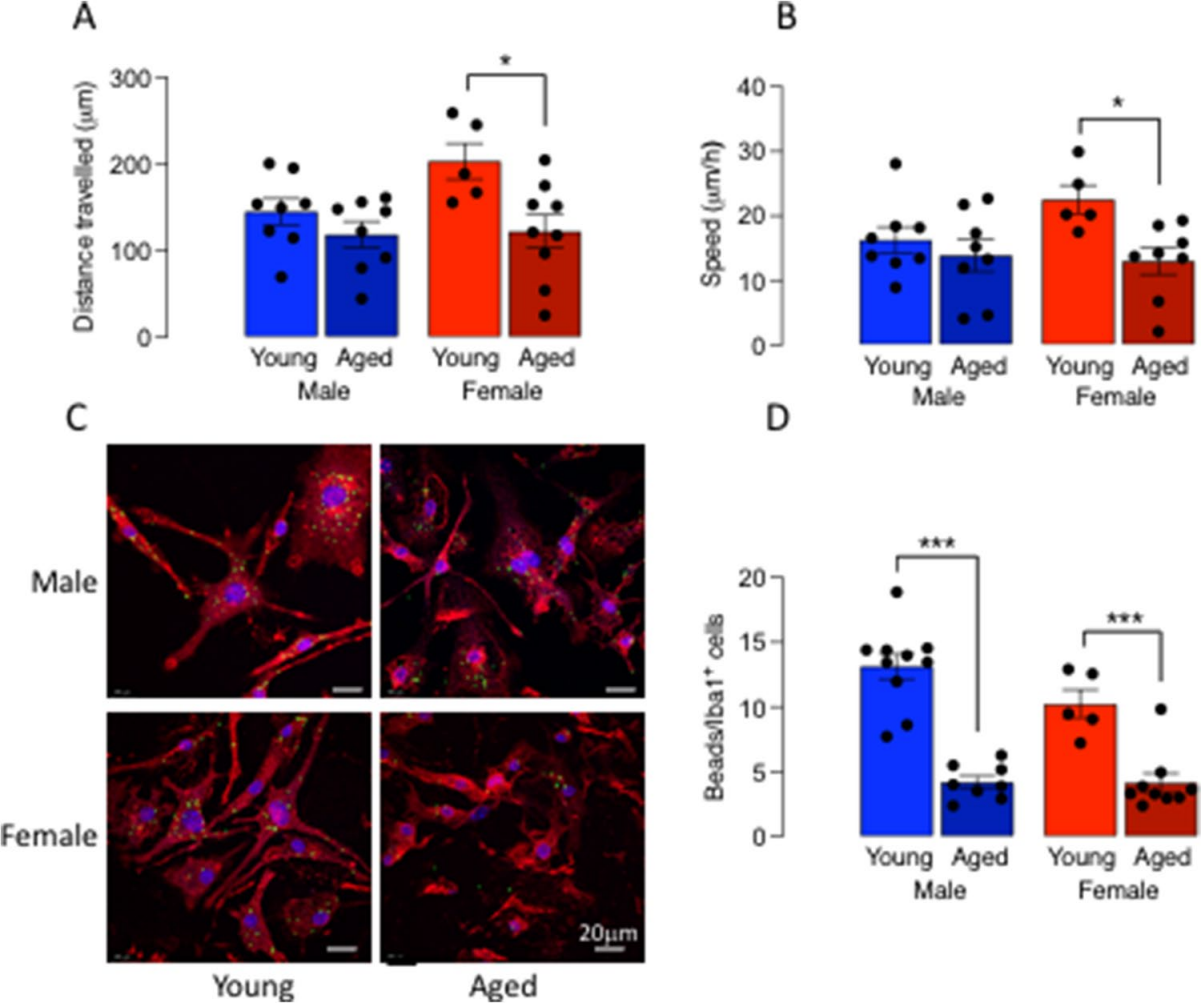
Age affects microglial motility in a sex-dependent manner. Microglia were prepared from young (4–5-month-old) and aged (17–22-month-old) mice as described in “Materials and Methods.” **A** Cell motility, assessed using the scratch wound-healing assay, revealed that the mean distance travelled by microglia (**A**) and the speed of movement 8 h post-injury (**B**) were significantly affected by age (*p* < 0.01 and *p* < 0.05, respectively), and post hoc analysis indicated that both measures were significantly decreased in microglia from aged, compared with young, female mice (*p < 0.05). **C**, **D** Analysis of uptake of fluorescently labelled beads revealed a significant main effect of age (*p* < 0.001). Post hoc analysis indicated that uptake was significantly decreased in microglia from aged male mice compared with young male mice and similarly in microglia from female mice (***p < 0.001). Data are expressed as the mean ± SEM (*n* = 5–10)

We assessed cell motility and migration using the scratch wound-healing assay and show that the distance travelled by microglia 8 h following the scratch injury, and the speed of movement was significantly affected by age (*p* < 0.01 and *p* < 0.05 respectively; Fig. 4A, B). There was a significant decrease in distance travelled in microglia from aged, compared with young, female mice (*p < 0.05; Fig. 4A) but no effect of age in microglia from males. Similarly, the speed of movement of microglia from female mice, but not male mice, was significantly reduced by age (*p < 0.05; Fig. 4B).

**Fig. 5:**
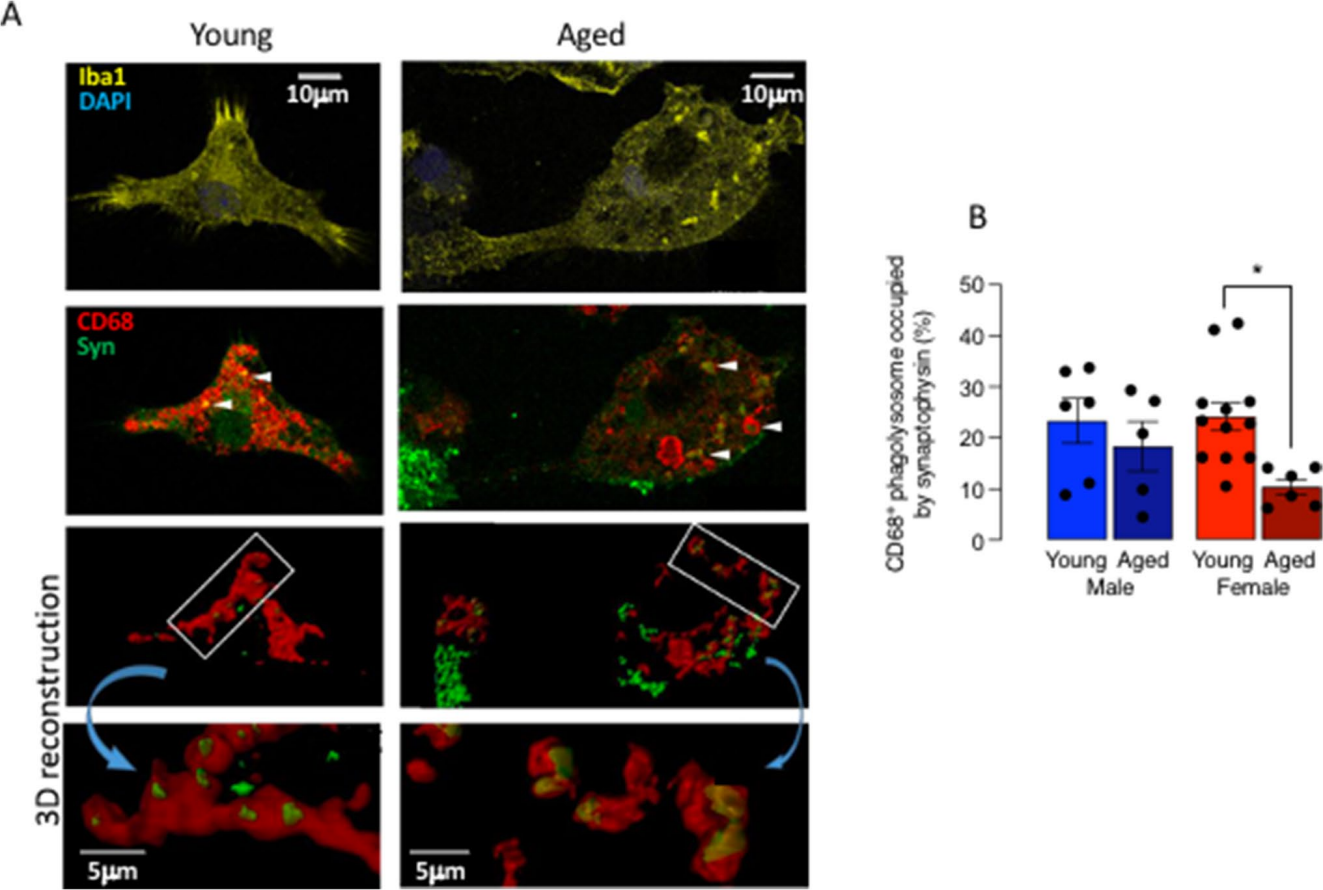
The age-related decrease in phagocytosis of synapses is confined to females. Microglia were prepared from young (4–5-month-old) and aged (17–22-months old) mice as described in the “Materials and Methods.” **A** Representative microphotographs and associated 3D reconstruction of confocal z-stacks for microglia isolated from young and aged mice following incubation (24 h) with synaptosomes. Iba1^+^ microglia (yellow) stain for CD68^+^ phagolysosomes (red). White arrow heads highlight synaptophysin (syn) staining (green) within CD68^+^ phagolysosomes (red). **B** CD68^+^ phagolysosome volume occupied by syn^+^ material (expressed as a % of total CD68.^+^ phagolysosomes) was significantly affected by sex (*p* < 0.05) and post hoc analysis indicated that this was significantly decreased in microglia from aged female mice compared with young female mice (*p < 0.05) Data are expressed as the mean ± SEM (*n* = 6–12)

We then assessed uptake of fluorescently labelled beads as one measure of function and found that there was a significant main effect of age (Fig. 4C, D) with post hoc analysis indicating that uptake was significantly decreased in microglia from aged male mice compared with young male mice and similarly in microglia from female mice (***p < 0.001; Fig. 4D). To further probe the age-related change in phagocytosis, we assessed engulfment of synapses by evaluating the proportion of CD68^+^ phagolysosomes occupied by synaptophysin. Figure 5A illustrates the 3D model system used to assess changes in synaptophysin uptake into phagolysosomes. Statistical analysis indicated that there was a significant main effect of sex (*p* < 0.05) with post hoc analysis indicating that there was a significant decrease in synaptophysin in CD68^+^ phagolysosomes in microglia from aged female mice compared with young female mice (*p < 0.05; Fig. 5B). Age did not affect phagocytosis of synapses in microglia from males.

## Discussion

The significant finding presented here is that sex has a marked impact on the effect of two different stressors on microglia. The data show that IFNγ  + Aβ, and also age, affect microglial metabolism and function; the evidence suggests that both stressors exert a greater effect on cells from female mice compared with cells prepared from male mice.

We have reported that IFNγ  + Aβ increases ECAR in cultured microglia from neonates [17]. The novel aspect of the present study is that there is a sex-related difference in metabolism in response to the stimulus; indeed, the evidence suggests that microglia from male mice responded minimally to IFNγ  + Aβ in terms of a metabolic shift, whereas a clear shift to glycolysis occurred in microglia from aged female mice. Similarly, the inducible form of PFKFB3 was enhanced to a greater degree in IFNγ  + Aβ-treated microglia from females compared with males and this is also broadly consistent with previous data that showed a positive correlation between glycolysis and PFKFB3 [13, 17].

LPS, like IFNγ  + Aβ, has been shown to increase glycolysis in primary microglia [25] and in BV2 cells [26, 27] although sex differences were not reported. It has been shown that sex impacts on LPS-induced [28] and IFNγ-induced [29] inflammatory cytokine release from microglia although in these studies release was greater in microglia isolated from the brain of P0/P1 male rats compared with females.

Nair and colleagues reported that the LPS-induced switch in metabolism towards glycolysis in primary microglia was driven by mitochondrial fission which caused mitochondrial fragmentation and inhibiting mitochondrial fission reversed the metabolic change and normalised the mitochondrial membrane potential [25]. These data point to a disruption in mitochondrial dynamics as a key factor in driving change. The present findings provide evidence of an age-related change in mitochondrial disruption but show that this is sex-related. Analysis of oxidative metabolism indicated that age triggers microglia from male mice to increase OCR and production of ATP; these changes do not occur in microglia from aged female mice and, indeed, the measures are markedly reduced in microglia from aged female mice compared with aged male mice. Thus, at least from the perspective of metabolism, it seems that microglia from male mice are better able to withstand the impact of the acute IFNγ + Aβ-induced stress and the chronic stress induced by age.

In comparing the effect of a stimulus in microglia from neonatal mice and adult mice, it is important to consider that the cells may respond differently. To check this, we compared the effect of IFNγ alone in 3 populations of cells, microglia from neonatal mice, microglia from young and aged mice, and microglia from wildtype and APP/PS1 mice. IFNγ increased mRNA expression of iNOS and TNFα in each case albeit with greater responses in microglia from aged, compared with young mice, and microglia from wildtype, compared with APP/PS1 mice (Supplementary Fig. 3).

One of the characteristics of microglia from APP/PS1 mice is that they are glycolytic [17, 22] and we recently established that this metabolic change was particularly notable in microglia from female APP/PS1 mice [22]. A second characteristic is that phagocytosis of Aβ was attenuated in APP/PS1 mice, accompanying the increased accumulation of Aβ [17, 22] and this was specific to female mice [22]. We considered that this might arise because of the metabolically inefficient glycolysis which yields only 2 molecules of ATP per glucose molecule compared with 32–36 ATP molecules produced by mitochondrial metabolism. The present data show that phagocytosis of latex beads was decreased with age in microglia from both males and females. However, the age-related engulfment of synapses was reduced only in microglia from female mice, suggesting that the more advantageous metabolic profile in microglia from males underpinned this maintenance of function.

Microglia are responsible for phagocytosis of not just synapses but also cell debris, myelin, and pathogens; different mechanisms have been ascribed to these, and it is also known that shape impacts on phagocytosis [30]. It has been reported that phagocytosis of latex beads is inhibited by an antibody directed at milk fat globule epidermal growth factor 8 (MFG-E8), suggesting its involvement in the process [31]. In contrast, phagocytosis of synaptosomes is dependent on the complement pathway and, in models of AD, inappropriate engulfment of synapses by microglia is responsible for synapse loss [32].

Several studies from this lab have demonstrated compromised function in glycolytic microglia with evidence of IFNγ-induced [33], age-related [13], and Aβ-related [17, 22] glycolysis linked with reduced phagocytosis. Here, we show cell motility is also affected by a change in microglial metabolism and specifically show a deficit in microglia from female mice which do not seem to have the capacity to withstand the stress associated with age.

The key message is that both acute and chronic stress, induced by IFNγ  + Aβ or age, affect microglial metabolism and function in a sex-dependent manner and that cells from females are more vulnerable to these stressors. The findings underline the importance of evaluating sex as a variable in studies on microglia.

## Supplementary Information

Below is the link to the electronic supplementary material.

Supplementary file1 (DOCX 6108 KB)

## Data Availability

All data are available on request from the corresponding author.
