# Supplementary material for: Acute Stress, Induced by IFNγ + Aβ, and Chronic Stress, Induced by Age, Affect Microglia in a Sex-Specific Manner
Source: Mol Neurobiol. 2023 Feb 14;60(6):3044–53. doi: 10.1007/s12035-023-03235-9 (PMC10122617; doi:10.1007/s12035-023-03235-9)
Supplement: Supplementary file 1 — Supplementary file1 (DOCX 6108 KB) [file 12035_2023_3235_MOESM1_ESM.docx]

**SUPPLEMENTARY FIGURES**

**
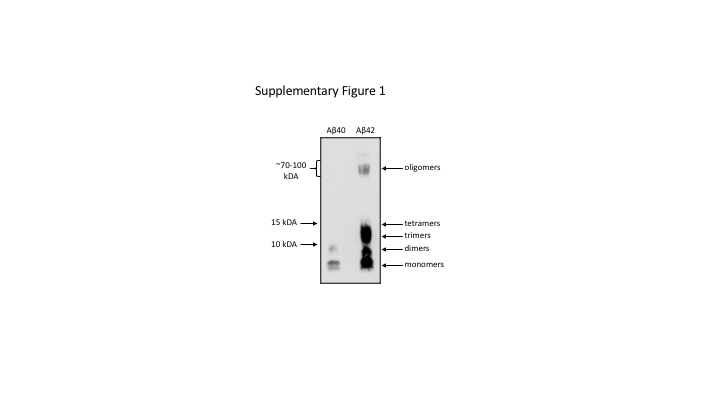
**

**Supplementary Figure 1**

To prepare Aβ which was used for treating microglia, lyophilized Aβ_1-40_ and Aβ_1-42_ peptides were dissolved in HPLC grade water to give a stock solution (6mg/ml). This was diluted (to

a final concentration (1mg/ml) using sterile PBS and aggregated (24 h, 220 rpm, 37°C). The composition of the resultant preparation was assessed by enhanced Thioflavin T binding and contained oligomers and fibrils.

**Supplementary Figure 2**

To assess microglial purity, cells were isolated as described in Methods, stained with a Hoechst/Propidium Iodide Stain (ThermoFisher Scientific, 62249, P3566), PE/Cy7-CD11b (Biolegend, 101215) and Alexa-fluor-647-GFAP (BD Bio, 561470) antibodies and assessed by flow cytometry (LSR Fortessa, BD Biosciences). Data were acquired using Diva software (BD Biosciences) and analyzed using FlowJo software. A,B. A. Single cells were gated using side scatter (SSC-A) vs. forward scatter (FSC-A) and for viability using Hoechst/Propidium iodide stain (B). C. The dot plot shows the CD11b stain on the x-axis and GFAP on the y-axis.

**
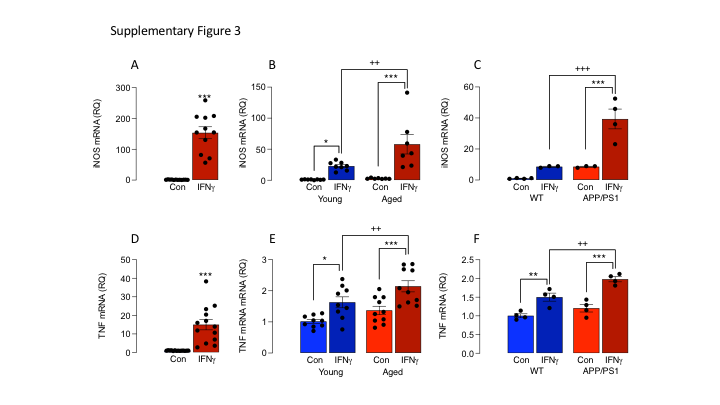
** **Supplementary Figure 3**

To compare the responses of microglia from mice of different ages, mRNA expression of iNOS (A-C) and TNFα (D-F) was assessed in cells (prepared as described in the Methods) from neonatal mice (A,D), young (4-5 months) and aged C57BL/6 mice (16-18 months; B, E) and 16-18 month-old transgenic mice that overexpress amyloid precursor protein (APP) and presenilin 1 (PS1; APP/PS1 mice) and littermate control wild type (WT) mice (C.F). RNA was isolated and assessed in cells as described for PFKFB3 mRNA (see Methods) using predesigned Taqman gene expression assays (TNFα (Mm0043258_m1); NOS2 (Mm00440502_m1; Applied Biosystems, Warrington, UK)) with β-actin as the endogenous control and gene expression was calculated as described for PFKFB3 mRNA.

**Supplementary Figure 4**

Microglial morphology was assessed in cells from neonatal (A), young (4-5 months; B) and aged (16-18 months; C) C57BL/6 mice. Microglia were washed, fixed in 4% PFA, washed again in PBT (PBS+1% Triton X-100), blocked (1 h; PBT+3% BSA) and then incubated with rabbit anti-Iba1 (19-7141, Wako, Japan 1:1000; overnight; 4^o^ C) as described in the Methods. After a further wash, cells were incubated with Alexa Fluor® 546 donkey anti-rabbit IgG (1:1000; 2 h; room temperature, A10040), mounted to enable images to be acquired and assessed in ProLong Gold with DAPI (P36941, Thermo Scientific, US). Images (8 fields; 40X magnification) were acquired with a Zeiss AX10 Imager A1 microscope.
